# Dual-process model of courage

**DOI:** 10.3389/fpsyg.2024.1376195

**Published:** 2024-03-22

**Authors:** Aakash A. Chowkase, Fabio Andrés Parra-Martínez, Mehdi Ghahremani, Zoe Bernstein, Gabrielle Finora, Robert J. Sternberg

**Affiliations:** ^1^Department of Psychology, University of California, Berkeley, Berkeley, CA, United States; ^2^Department of Education Reform, College of Education and Health Professions, University of Arkansas, Fayetteville, AR, United States; ^3^Department of Counseling, Higher Education Leadership, Educational Psychology, and Foundations, Mississippi State University, Starkville, MS, United States; ^4^Department of Psychology, College of Human Ecology, Cornell University, Ithaca, NY, United States; ^5^Division of Nutritional Sciences, College of Human Ecology, Cornell University, Ithaca, NY, United States

**Keywords:** courage, dual-system theory, type I thinking, type II thinking, noble purpose, approach-avoidance conflict, expectancy-value theory, risk assessment

## Abstract

Courage is one of the most significant psychological constructs for society, but not one of the most frequently studied. This paper presents a process model of courage consisting of decision-based pathways by which one comes to enact a courageous action. We argue the process of courage begins with a trigger involving an actor(s) and a situation(s). The actor(s) then engage(s) in four key assessments concerning (a) immediacy of the situation, (b) meaningfulness, value, and relevance to the actor, (c) adequacy of efficacy to act, and (d) decision to act with courage. The central component of this process entails an approach-avoidance conflict involving assessments of perceived risks and potential noble outcomes of acting with courage. The decision to act may result in courageous actions assuming it satisfies the four elements: intentionality, objective and substantial risk, a noble purpose, and meaning in time and place. Courageous actions have consequences. Finally, the consequences shape the actors’ experience, which feeds into the trigger, closing the loop. Potential moderators of the courage process as well as potential tests of the model have been discussed.

## 1 Introduction

“Courage is not the absence of fear, but rather the assessment that something else is more important than fear” – Roosevelt (1932).

Courage is often a deliberate assessment of a trigger and an analyzed decision on how to proceed toward a noble purpose in the face of personal risks (Rate et al., 2007). Courage is one of the most significant psychological constructs for society, but not one of the most frequently studied. Sternberg (2022b) has suggested that, for humanity, courage is the most important gift of all. For over a year now (March 2024), people in Iran have been protesting against the theocratic regime for wider freedoms and women’s rights (Alkhaldi and Ebrahim, 2022; Reuters, 2022a). Similarly, despite the rising number of killings of activists in Colombia, courageous social leaders resist threats in their quest to defend people’s basic human rights after five decades of armed conflict (Prem et al., 2018; Llanes et al., 2022). Another similar case is that of the ongoing war between Ukraine and Russia, in which Ukrainian citizens and the Ukrainian army are fighting not just for their country’s sovereignty, but also for survival (Hook, 2022). On another note, today, many countries, including the United States, are faced with threats to their democracies (Albright, 2018; Mounk, 2018; Ziblatt and Levitsky, 2018; Applebaum, 2021; Ben-Ghiat, 2021). Without the courage of the citizens in these countries, those democracies may well be lost.

Not only is courage important at the level of a country. It also is important in one’s daily life. Examples are voicing opinions or standing up for oneself or one’s colleagues who might be facing injustice in the workplace (Schilpzand et al., 2015).

But what, exactly, is courage? What are the steps leading to courageous actions, and what kinds of factors lead people to be more or less likely to display courage? In this article, we build on existing scholarship on courage and propose a novel dual-process model that describes courage as a deliberate, multi-stage process involving numerous decision-based pathways by which one comes to enact a courageous action–or not.

## 2 What is courage?

### 2.1 The courageous actor

Oftentimes, courage is understood in terms of courageous people–individuals who are labeled either as courageous or not (Pury and Starkey, 2010; Rate, 2010). However, courage is not just a trait, but also a virtue, a skill, and an attitude toward a given situation (Comte-Sponville, 2002; Jacobson, 2005). It can also be a trait or a state (Sternberg, 2022a). Courage is considered to be one of the six core virtues contributing to goodness among humans across nations, cultures, and religious beliefs (Peterson and Seligman, 2004). It is also one of the most universally respected virtues, as it is essential in practicing all other virtues (Comte-Sponville, 2002). Understanding courage as a virtue implies that people with noble intentions can perform courageous acts. Similarly, when courage is understood as a skill as well as an attitude, one can learn and practice courage. Moreover, one who repeatedly performs courageous behaviors is typically perceived as possessing the trait of courage (Rate et al., 2007; Pury and Starkey, 2010; Rate, 2010). The Iranian protests’ growth over time indicates that courage is a virtue as well as a learned behavior that many can practice by transforming their noble intentions into actions.

### 2.2 The courageous act

Courage is also often understood through its enacted aspect–the courageous act. Courage can be manifested in a variety of ways and situations, from local and possibly daily instances to large-scale, possibly once-in-a-lifetime global actions (Pury and Lopez, 2010; Koerner, 2014). Perhaps the prototypical instance of courage would be resisting the abuse of power. One can see this resistance in daily life as well as on a larger scale. On a local level, defiance of abuse of power can be exemplified by an individual confronting a bully at the workplace (workplace courage; Sekerka and Bagozzi, 2007; Schilpzand et al., 2015). On a larger, more international scale, defiance of abuse of power can be seen in civil unrest against governments perceived as unjust.

Speculations in the Western world on the nature of courage date back at least to ancient Greece. Of particular relevance would be philosophical accounts by Epictetus (1983), Aristotle (1985), Plato (1987), Aquinas (1960), and Sartre (1967). In the Eastern world, Confucius (1992) also wrote about courage. A comprehensive philosophical review of the concept of courage can be found in Putman (2010).

Pury and Lopez (2010) compiled the reflections of modern psychologists on the notion of courage. Lopez et al. (2010) examined folk conceptions of courage and concluded that an essential element of courage is the presence of personal risk. Perhaps the most accepted definition of courage was devised by Rate et al. (2007), Rate (2010); see also Rate and Sternberg (2007). By examining implicit theories (folk conceptions) of courage, they characterized courage (a) as representing an act that is willful and intentional; (b) that is executed after reflective and mindful deliberation; (c) that presents an objective, substantial risk to the individual; (d) that is motivated primarily, although not necessarily exclusively, in the service of a worthy purpose or a noble good; and (e) that is enacted despite the challenge of feeling fearful. Rate (2010), in subsequent research presenting an explicit theory based on the collection of empirical data, suggested as necessary elements: intentionality, objective and substantial risk, and a noble purpose. We now discuss each of these three aspects in greater detail below.

#### 2.2.1 Courageous acts are intentional and deliberate

Consistent with Rate and colleagues’ definition (Rate et al., 2007; Rate, 2010), we maintain that courageous acts are intentional and deliberate. Although some courageous acts may occur almost instantaneously, for example, saving someone from drowning in a pool, many courageous acts are pursued intentionally but emerge out of deliberate thinking (Shelp, 1984; Rate et al., 2007; Pury and Starkey, 2010; Rate, 2010). A dual-system theory (Kahneman, 2011) can be used to explain these two types of responses. In urgent situations, time is a critical factor, as it can drastically affect the outcome. Such situations may initiate a Type I or intuitive response (e.g., immediately jumping into a pool to save a drowning person). Other situations that afford deliberation may invoke a slower and more analytic Type II response. In this article, we focus primarily on Type II thinking, conceptualizing courage as an intentional and deliberate process.

#### 2.2.2 Courageous acts require risk management

Most definitions and theoretical models of courage view courage as a goal-directed process that involves weighing potential personal risks and noble or morally worthy outcomes before taking action (MacIntyre, 1981; Shelp, 1984; Worline, 2004, 2012; Hannah et al., 2007; Rate et al., 2007; Quinn and Worline, 2008; Lopez et al., 2010; Rate, 2010; Koerner, 2014; Schilpzand et al., 2015). Research has consistently shown that perceptions of the potential risks versus benefits of a particular action influence the decision to take risks (Moore and Gullone, 1996; Foster et al., 2009; Zhang et al., 2016). This risk–benefit analysis can be explained through the concept of approach-avoidance conflict (Lewin, 1931; Miller, 1944; Dollard and Miller, 1950), as discussed later in the description of the proposed dual-process model of courage.

#### 2.2.3 Courageous acts promote a noble purpose

A courageous act involves risk management, but courage cannot be reduced to calculating risks and benefits (Shelp, 1984; Rate et al., 2007; Rate, 2010). Courage is primarily motivated by a desire to bring about a worthy or noble purpose (Shelp, 1984; Walton, 1986; Woodard, 2004; Rate et al., 2007; Woodard and Pury, 2007; Rate, 2010). Courage entails at least as much interest in the welfare of others and of society as it does interest in one’s own welfare. In other words, courage cannot primarily originate in the pursuit of self-interest or fame. Rather, truly courageous actors act out of noble motivations (Shelp, 1984; Walton, 1986; Woodard, 2004; Rate et al., 2007; Woodard and Pury, 2007; Rate, 2010). Although “noble” can mean several things, researchers often use it to mean prosocial, moral, or virtuous (Shelp, 1984; Rate, 2010; Howard and Alipour, 2014; Howard et al., 2017).

However, it is worth noting that many people consider actions they have taken only for their own benefit but not directly for others’ welfare (e.g., going on a roller coaster) as courageous (Finfgeld, 1999; Pury et al., 2007; Muris, 2009; Pury and Saylors, 2017). Pury and Starkey (2010) have observed that, at times, individuals can exhibit what might be characterized as *bad courage*, or the courage to pursue an action that may seem noble to oneself, but that is morally misguided and possibly reprehensible. That is, the person convinces themselves that they are acting for a good cause when they are actually acting for a bad cause. For example, terrorist attacks are sometimes justified as serving a noble purpose, when, in fact, they tragically result in the disruption, ruination, and often, loss of innocent lives (Silke, 2004). Similarly, “bad courage” can be seen through state terrorism, which is often rationalized as a necessary evil in order to protect the state, yet, most often amounts to giving dictators unrestricted power. However, we focus on courage that is essentially directed at achieving a noble goal. Leaving out the pursuit of a noble goal from the construal of courage allows for validating and even justifying evil acts as courageous and, therefore, societally accepted.

#### 2.2.4 Courageous acts are viewed in context

In addition to intentionality, objective and substantial risk, and a noble purpose, we posit that courage is contextual and the designation of an act as “courageous” can vary, depending on the time, culture, and place (Shelp, 1984; Pury et al., 2007; Howard and Cogswell, 2019; Sternberg, 2022b). What is considered courageous by some may not be seen the same way by others. For example, Rosa Parks’s refusal to give up her seat to a White man on a bus in Montgomery, Alabama in 1955, is now widely accepted as a courageous action. It served the noble cause of standing up to racism; it was an intentional, deliberate, and risky action; and it was radical in a time when people of color were expected to give up their seats to White persons and were not permitted to sit near White persons. However, Rosa Parks’s act was not seen by all as courageous when it took place in 1955. At that time, some people almost certainly viewed Rosa Parks as foolish in her ability to manage risk and even as wrong or evil because they went against the “natural” order of society. In today’s world, it is not as surprising to see women taking a political stance. Many of them are leading various political movements, such as the ongoing protests by women in Iran (Alkhaldi and Ebrahim, 2022). Modern day examples that demonstrate courage is contextual include Caitlyn Jenner’s public gender transition and, on the other hand, Kim Davis’s refusal to issue same-gender marriage licenses. Their actions are contextually perceived as courageous by some, but not by others. The perception of courage is significantly based on the value the perceiver sees of the goal and, not just, the risk of the action (Pury et al., 2024).

The courageousness of an act also depends on the comparison group. Pury et al. (2007) provided empirical evidence distinguishing *general courage* (actions that would be courageous for anyone to take) and *personal courage* (actions that are courageous only in the context of an individual’s life). On the one hand, actions displaying high levels of general courage are often perceived as courageous by others. On the other hand, actions demonstrating high levels of personal courage may be regarded as particularly noble by individuals familiar with the person but not necessarily by others.

In summary, we propose that courageous acts are intentional and deliberate endeavors that involve significant risk management, serve a noble purpose, and whose outcomes have an impact on the context, given the time and place. This definition may help to assess courageous acts as an overlap of all of the above-mentioned elements. However, this understanding is not prescriptive; rather, it is only descriptive. We believe it is impossible to evaluate the long-term outcomes of a courageous action in the immediate present. Nevertheless, examples from the past can inform our understanding of courage. By looking at past examples, we can identify and describe whether an action was courageous in terms of intentionality, deliberation, risk management, a noble purpose, and impact in the context of time and place.

Pury and Starkey (2010) proposed two ways of studying courage: as an accolade and as a process. As an accolade, courage is typically viewed as rare, lofty, and worthy of societal acknowledgment. On the other hand, courage is seen as a process by which people overcome subjectively felt risks for compelling reasons. Pury and Starkey (2010) further argue that the accolade perspective on courage offers information about people who have performed exceptionally well and how they are different from the rest. However, courage when studied as a process offers information about ways in which people come to act courageously.

Although in this section we have described courage as an act, the account presented above makes it clear that courage is also often believed to be the result of a deliberate process (Rate et al., 2007; Pury and Starkey, 2010; Rate, 2010), thereby allowing courage to be investigated as a psychological process involving several connected steps and decisions. However, few scholars have proposed and validated detailed process models of courage (but see, e.g., Hannah et al., 2007; Sekerka and Bagozzi, 2007; Schilpzand et al., 2015). Although there is ample scholarship that focuses on the elements of courage, there is still a dearth of studies that clarify and organize the complex concept of courage into a structured framework of processes. Therefore, in this article, we propose a dual-process model of courage. By delineating the process into various stages and assessments, our model aims to provide clarity on how courage unfolds and what factors influence courageous behavior. Moreover, understanding the process of courage can have practical applications in various domains, including psychology, education, organizational behavior, and leadership development. Our model offers insights into how individuals evaluate and navigate challenging situations, make decisions under uncertainty, and manage risk in pursuit of noble goals. This understanding can inform interventions, training programs, and organizational policies aimed at fostering courage. By identifying critical assessment points and exit paths, the model offers insights into factors that may facilitate or inhibit courageous behavior. This predictive aspect may enable researchers and practitioners to anticipate when and how individuals are likely to act courageously, as well as to develop strategies for cultivating courage in individuals and organizations. In proposing this new model, we integrate insights from various disciplines, including psychology, ethics, and decision theory, into a unified framework. This interdisciplinary approach allows for a comprehensive understanding of courage that goes beyond traditional disciplinary boundaries. By synthesizing existing research findings and theories, our model potentially contributes to a more holistic understanding of courage and its determinants. Although our definition of courage focuses on the pursuit of a noble goal, the process model we propose can possibly apply to other types of courage (e.g., bad courage, personal courage) where the focus on pursuing a noble goal is not considered essential.

To facilitate a detailed explanation of the model, throughout the article, we use the example of ongoing protests in Iran against the brutalities of the autocratic regime. For context, a series of protests and civil unrest against the Iranian regime was sparked in September 2022 after the death of 22-year-old Mahsa Amini, who was arrested and beaten by the ironically labeled “morality police” for violating Iran’s mandatory dress code for women (Alkhaldi and Ebrahim, 2022; Reuters, 2022a). In response to the protests, the Iranian government has been using brutal and utterly cruel security forces to curb the protests. By April 2023, more than 530 protestors had lost their lives, including about 70 minors; and 22,000 have been jailed (Karimi and Gambrell, 2023). Despite the brutal repression and the enormous risk to their lives, resistance has been growing in Iran, with the hope, on the part of the resistors, of seeing a positive change. The courage demonstrated by many Iranians, especially women and students, is evident in this example. Therefore, we refer to this example throughout this article to elaborate on the proposed process model of courage.

## 3 Courage as a process

We are not the first to present a process model of courage. However, our contribution is novel in that it draws on modern conceptions of Type I (fast and intuitive) and Type II (reflective, deliberative) thinking. Hannah et al. (2007) devised a Subjective Experience of Courage model, in which they set forth the notion that a courageous mindset promotes an individual’s skills and systems to combat fear and replaces that fear with a courageous act. Sekerka and Bagozzi (2007) devised a process-orientation theory for conceptualizing moral courage. Their theory highlights personal choice as an indispensable component, as well as the presence of an ethical dilemma that serves as the impetus for courageous action. Schilpzand et al. (2015) evaluated courage from a workplace perspective and set forth a two-pronged model, beginning with an assessment of individual responsibility in relation to a challenge and subsequently factoring in the potential social consequences of acting.

Our model expands upon its predecessors and broadens the scope of the situations viewed as potentially eliciting courageous acts. It further takes into account how one deliberately engages in moral considerations and risk assessment to approach or avoid situations needing courage. Our model aims to emphasize the role of individual agency as well as the recursive nature of the process of courage to represent the dynamic decision-making process applicable to a variety of situations. In this regard, our model is different from models that focus only on workplace dynamics (e.g., Sekerka and Bagozzi, 2007; Schilpzand et al., 2015) and from models that focus only on some attributes of courage, such as risk-taking and goal value (e.g., Pury et al., 2024).

## 4 A dual-process model view of courage

Courage has traditionally been thought to be the result of attributes such as integrity, honor, valor, independence, a sense of duty, selflessness, loyalty (Park et al., 2004), bravery, persistence, and vitality (Pury and Kowalski, 2007). However, we now view courage as a multi-stage process that involves assessing a situation, as driven by both prosocial emotions and cognitive recognition of an opportunity to right a wrong or potential wrong.

**Figure 1:**
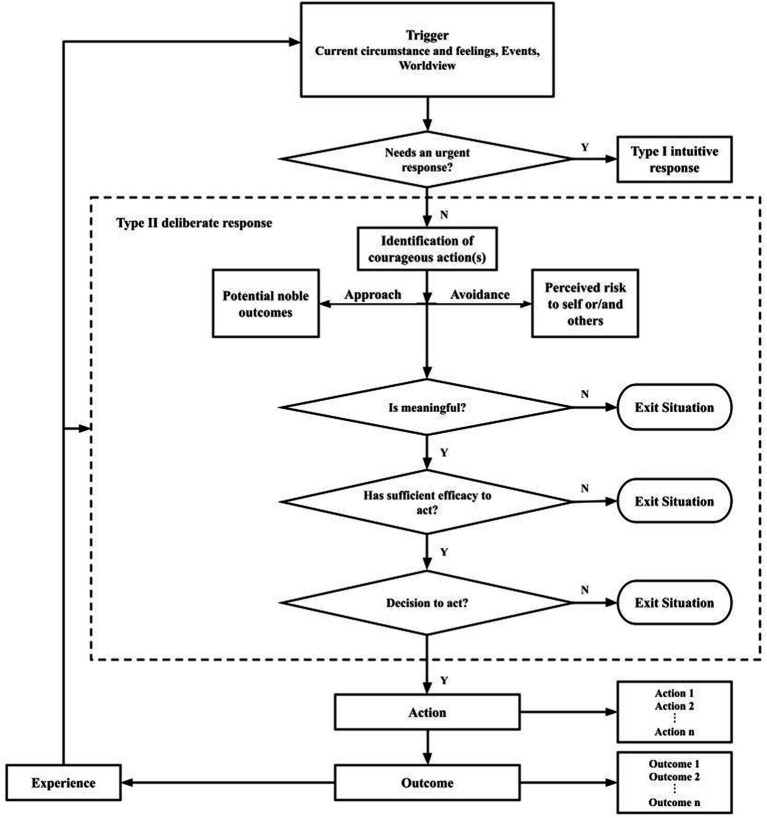
The dual-process model of courage.

In this article, we aim to extend current understandings of the psychological construct of courage. In Figure 1, we propose a dual-process model consisting of various decision-based pathways by which individuals come to enact a courageous action, or not. This model can be used to examine and influence the decision-making process underlying courageous actions. It also can help to understand how and why an individual in a particular challenging situation proceeded with their chosen action.

According to our dual-process model of courage, courage commences with a trigger that involves an actor or actors and a particular situation. This actor is tasked with making four critical assessments, encompassing (a) the immediacy of the situation; (b) its meaningfulness, relevance, and value to the actor; (c) one’s self-efficacy to act; and (d) the decision to act with courage.

Courage entails an approach-avoidance conflict, guided by moral considerations in pursuit of a noble goal, that necessitates deliberate assessments of perceived risks and potential morally commendable outcomes. The decision to act may lead to courageous actions, provided the decision aligns with the elements of courage in the previously mentioned definition: intentionality, deliberation, risk management, noble intent, and impact in a specific time and place. These actions can yield various outcomes or consequences, and these outcomes, in turn, can shape the actors’ experiences, thus completing a feedback loop. Notably, the dual-process model encompasses four exit paths, one corresponding to each assessment point.

### 4.1 Trigger

Like many behavioral models, we propose that the process of displaying courage commences with a trigger. Courage emerges in response to situations characterized by significant personal risks, threats, and obstacles, as noted in previous studies (Cavanagh and Moberg, 1999; Harris, 2001; Lopez et al., 2010). This courage-inducing trigger can take various forms, including the actor’s current circumstances (e.g., poverty), their emotional state (e.g., a sense of outrage at injustice), or a specific event (e.g., a fire out of control). It is essential to emphasize that the trigger need not necessarily be life-threatening; it also can be rooted in the actor’s worldview, often influenced by their previous experiences, personal histories, and societal norms. In our example of the Iranian protests, Mahsa Amini’s death served as the trigger that motivated Iranian citizens to confront the injustices committed by their government, and then to resist police brutality.

A trigger can be situational and have the potential to elicit a wide range of emotions, including fear, anger, disgust, and compassion. Many scholars in the field of courage, such as Shelp (1984), Peterson and Seligman (2004), Rachman (2004), Woodard (2004), Woodard and Pury (2007), Rate et al. (2007), and Kilmann et al. (2013), have suggested a close link between courage and fear. Pury et al. (2007) have demonstrated that fear and confidence (self-efficacy) are distinctively related to general courage (actions considered courageous for anyone) and personal courage (actions courageous within the context of an individual’s life).

Their research reveals that actions demonstrating high levels of general courage are characterized by substantial confidence and minimal fear. Conversely, actions displaying high levels of personal courage involve acting despite fear, adversity, and individual limitations. Although there is some evidence suggesting that a small group of people may not experience fear, even in high-risk situations (Rachman, 2004), empirical support for fear as a typical, though not essential, component of courage appears relatively weak. Nonetheless, it is important to note that fear can be diminished through repeated acts of risk-taking, as seen in contexts such as military training and practice (Walk, 1956), or when individuals have adequate resources to cope with adversity (Rachman, 2004). Therefore, further research is required to gain a deeper understanding of the intricacies of the relationship between fear and courage.

### 4.2 The first assessment: urgency of the situation

Once triggered, the initial assessment in the courage process may not necessarily be well-thought-out. Instead, it often represents an intuitive and immediate assessment of the situation to determine if an urgent response is required. For instance, individuals faced with dire circumstances, like a house fire or a child drowning, may not have the luxury of time to reflect on the situation or to consider the consequences of their actions. In such cases, they must rely on instinct and act without much deliberation. However, in less immediately challenging situations, individuals may have the opportunity to take some time to process the situation and act more deliberately.

In the dual-system theory (dual-process theory) proposed by Kahneman (2011), human cognition is regulated by two distinct systems or modes: System I for fast, intuitive, and effortless thinking, and System II for slower, analytic, and effortful thinking. Although some acts of courage may indeed arise intuitively due to the urgency of the situation, most courageous acts involve deliberation and reflection. In other words, although some intuitive courageous responses might result from the automatic and often unconscious operations of System I, such as rescuing someone from a burning building, the majority of acts of courage entail the controlled operations of System II. These controlled operations demand more significant cognitive effort (Kahneman, 2011), particularly in situations requiring courage, such as the ongoing situation in Iran. Therefore, our model primarily focuses on the controlled operations of System II. Nevertheless, this focus is not to downplay the role of intuitive courage in everyday life, especially in emergencies where Type II thinking would be too time-consuming. However, when the situation permits time for deliberation, we propose that the actor typically deploys Type II thinking.

### 4.3 Type II thinking: risk assessment, moral considerations, and approach-avoidance conflict

Arguably, the most important component of this model is Type II thinking (Kahneman, 2011) – that is, slower, analytic, and effortful thinking potentially involving several moral and risk considerations. On one hand, courageous actors show a willingness to assume substantial personal risk to pursue a noble goal (approach motivation), but on the other hand, they also consider the risks involved in such a pursuit (avoidance motivation). In this primarily cognitive process, the actors identify the courageous actions that potentially can help achieve the noble goal and the risks associated with potentially taking those actions; they then engage in deliberate moral reasoning, and perhaps a moral dilemma as to whether to engage with the situation.

The actor experiences approach-avoidance conflict and may deliberately engage in a personally satisfying resolution (Dollard and Miller, 1950). Here, the actor engages in three major systems: (a) a behavioral activation system, prompted by morally rewarding stimuli, such as the pursuit of a noble goal (approach motivation); (b) a fight-flight-freeze system, prompted by harmful or threatening stimuli, such as the risks involved (avoidance motivation); and (c) a behavioral inhibition system, prompted by negotiation between potential noble outcomes and perceived risks (approach-avoidance conflict) (McNaughton and Gray, 2000; Corr, 2013).

An approach-avoidance conflict arises when an actor is both drawn to and repulsed by the same trigger (Lewin, 1931; Miller, 1944; Dollard and Miller, 1950). Consequently, the System II decision the actor makes about approaching or avoiding the situation results from reaching a point of negotiation between the relative valences of perceived risks and potential noble outcomes that may arise out of acting courageously. For example, in the Iranian protests, approach-avoidance conflict involves conflict between potential risks to life and safety (avoidance) and the pursuit of a positive change in the lives of Iranian women (approach). The Iranian regime has repeatedly used various brutal and, indeed, savage methods to curb the protests, such as shutting down Internet and cellphone service, arresting journalists, performing mass detentions, and torturing captured protestors (France 24, 2022; Reuters, 2022a,b). Evidently, the risks of participating in the ongoing protests can be grave. However, the pursuit of a positive change in women’s lives seems at least as strong, at least for some Iranians, as the protests are still active.

A situation involving a greater perceived risk will involve a greater approach-avoidance conflict and will, therefore, demand greater courage. The anterior hippocampus area appears to be central in the processing of approach-avoidance conflict (Bach et al., 2014; O’Neil et al., 2015; see Ito and Lee, 2016, for a review). Bach et al. (2014) gave neurologically healthy participants a computerized task involving approach-avoidance conflict, as administered under three different levels of threat. When the likelihood of threat increased and so did the level of approach-avoidance conflict, the participants displayed greater avoidance behavior and behavioral inhibition. These changes in avoidance behavior were accompanied by changes in hippocampal activity. When the threat level increased, hippocampal activity also increased, suggesting the hippocampal area of the human brain played an important role in approach-avoidance conflict processing.

As is typical of System II thinking, the actors can be expected to engage in serial, conscious, and consequential decision-making (Kahneman, 2011). For example, approach-avoidance conflict may involve carefully assessing the relative values of available options and ascertaining, as well as one can, the price, probability, and magnitude of the consequences associated with various outcomes (Rolls and Grabenhorst, 2008; Quartz, 2009; Aupperle Robin and Paulus Martin, 2010). From a motivational perspective, two key determinants of choice are the relative values of various options and the expectancies of success (Eccles and Wigfield, 2002). The value component depicts the desirability of a particular goal (i.e., “Why would I engage in such an activity?”), whereas the expectancy component represents an individual’s beliefs about how well they will do in an activity (i.e., “Can I be successful in doing this activity?”). We discuss these two key considerations in the next two subsections.

### 4.4 The second assessment: value, meaningfulness, and relevance

When confronted with a situational trigger, individuals may face yet another assessment regarding whether or not the situation presents an adequately personally valuable and meaningful goal. An individual would possibly consider engaging with the situation as personally meaningful because it might help them get closer to a valued goal. A situation requiring courage can hold significance for individuals because it is closely intertwined with their own lives, falls within their circle of concern, and is morally commendable. For example, employees feel motivated to act courageously when the cause is meaningful to them and they feel a sense of personal responsibility (Schilpzand et al., 2015).

Individuals are more inclined to engage in a risky situation when it involves people, ideas, or moral principles about which they care deeply (Shelp, 1984). For example, many advocates for LGBTQ+ rights are members of the LGBTQ+ community themselves and draw upon their personal experiences of discrimination as motivation to champion this cause. The same principle applies to Iranian citizens and members of the diaspora who have taken to the streets to advocate for Iranian women’s rights because the issue directly affects their daily lives.

Actors can also attribute personal meaning to a situation even if it does not directly involve them or anyone in their immediate social circle. This sense of meaning may arise from their ability to expand their circle of concern. Driven by an elevated sense of interconnectedness, compassion, and empathy, actors can form connections with strangers, including members of outgroups, motivated by a focus on a superordinate identity and a recognition of common humanity (Chowkase, 2022, 2023; Chowkase and Watve, 2022).

From a virtue perspective, considerations of moral principles can also guide the decision-making process (Peterson and Seligman, 2004). On perceiving a challenging situation, an individual may recognize its moral and ethical importance. They may view the situation as an opportunity to act in accordance with their core moral ideals (Bredemeier and Shields, 2006; Schwartz et al., 2012). In doing so, the actor may reflect on their fundamental moral values, such as integrity, equity, and fairness, and consider how their potential acts in a given situation can exemplify these virtues and put their moral character into action (Peterson and Seligman, 2004; Lapsley and Carlo, 2014). Moreover, the individual may recognize that their actions can positively impact themselves, others, or the greater good. They then may feel that it is incumbent upon them to act accordingly (Brown and Treviño, 2014). Such a sense of duty and perceived ethical responsibility may motivate an individual to approach a challenging situation.

The extent to which an individual musters the courage to act may depend, in part, on the values the situation holds for them. In other words, if the actor perceives the challenging situation, their action in response, and its potential consequences as promoting personally valued goals, they are more likely to engage with the situation. Conversely, if they do not find the situation personally meaningful, they may opt to disengage from it, as indicated in Figure 1 with a stop symbol.

The greater the degree of meaningfulness the situation holds for the actor, the more likely they are to engage with it and exhibit courageous behavior. However, it is crucial to note that meaningfulness alone is not sufficient; the actors’ willingness to act courageously may also be influenced by their expectancy regarding the likely outcome and by their self-efficacy beliefs.

### 4.5 The third assessment: efficacy

In the process of displaying courage, the next assessment concerns beliefs about an individual’s ability to perform a courageous action and to achieve success in that action. Bandura (1977) defined outcome expectancy as a person’s estimate that a given behavior will lead to certain outcomes. If an actor does not believe in the possibility of a successful outcome, then the behavior leading to that outcome is unlikely to occur. Additionally, actors need self-efficacy or confidence in their ability to perform the action (Bandura, 1982). In this assessment, actors evaluate whether or not they believe they have the resources or skills needed to execute the action successfully. For instance, do they have confidence in their skills to act courageously? Do they think they have enough information, strategies, and tools to effectively engage in a protest? Considering the potential repercussions of joining the protest, are they still confident they can achieve success by joining?

Although specific beliefs about one’s ability and one’s likelihood of success are predictive of one’s behaviors, one specific form of self-efficacy belief may be most relevant to courage. Moral self-efficacy is one’s confidence in one’s ability to handle ethical problems (May et al., 2014). For courage to be enacted, the actors display high confidence in their ability to act morally in the situation and that they have a chance to succeed with their action(s). However, if actors lack sufficient moral self-efficacy in a situation, despite possessing strong approach motivation, they are likely to exit the situation. Cognitively, they may know what is moral in the situation, perceive the risks and potential noble outcomes well, and want to approach the situation. Yet, they may decide to leave the situation because they lack the confidence to act morally. This situation is indicated with a “stop” symbol in Figure 1. However, when the actors feel sufficiently self-efficacious about handling the situation and especially feel sufficiently confident to take a moral stand, they are likely to proceed to the next step.

In the case of protests in Iran, many Iranian people have shown exemplary self-efficacy–the confidence that they can win their fundamental rights. Thus, they have continued to resist the abusive power structure, regardless of violent threats from the regime (Rajvanshi, 2022).

### 4.6 The fourth assessment: decision to engage

After consolidating information from all the previous assessments, the actor arrives at the final assessment regarding whether to engage in the courage-demanding situation or not. When the actors sufficiently value engaging with the situation and have enough self-efficacy, and therefore, have the motivation to engage, they are likely to decide to engage in the situation and perform a courageous act.

When avoidance motivation or the fight-flight-freeze system dominates the processing of a conflict, the actors are more likely to cave in and avoid the situation, regardless of their desire to confront the situation. Importantly, avoidance is an active decision made to avoid potential adverse outcomes, such as getting arrested while protesting against the government.

In contrast, when approach motivation or the behavioral-activation system dominates the processing of the conflict, actors can be expected to engage with the situation, despite impending risks. Strong approach motivation may emerge in a situation such as the one in which Rosa Parks found herself on a bus in Montgomery, Alabama; despite her knowing that refusing to give up her seat most likely would lead to adverse outcomes, she remained seated. Her will to fight for her rights and the rights of people of color all across the United States trumped her fear of any impending consequences. She was fired from her job following the bus boycott and received death threats for years afterward.

In more recent times, heroic individuals such as Alexei Navalny, a prominent anti-corruption activist in Russia, and Narges Mohammadi, an Iranian human rights activist and Nobel laureate, have demonstrated courage similar to that of Rosa Parks and are facing brutal consequences for their courageous actions. Navalny’s actions are recognized as modern examples of unwavering courage against corruption, with some suggesting his name become synonymous with resilience and courage (Bazargan, 2024). He was recognized most distinctly for publishing corruption-exposing investigations against Putin’s regime (Chappell, 2024). The world mourns his passing on February 16th, 2024, with thousands of supporters attending his funeral in Russia on March 1st, 2024 (Hopkins, 2024). As evident in these examples, *approach*, like *avoidance*, is also an active decision and may involve sacrificing, possibly forever, personal safety, pleasures, or self-interest.

Most often, courage requires possessing and demonstrating strong approach motivation. For example, in the case of the ongoing protests in Iran, people’s courage lies in confronting the unjust and powerful system, despite risks and other impediments to action. In this case, approach motivation thrives on the potential noble outcomes the protests may yield, namely, gaining human rights for Iranian women. Other motivators are attaining better visibility for the women’s poor condition, gaining people’s support within and outside Iran, and possibly bringing about systemic change in the oppressive regime. When the actors lack the necessary level of relevant motivation, they are most likely to exit the situation without engaging with it any further. This situation is indicated in Figure 1 with a “stop” symbol.

This decision to engage also involves strategizing about how the courageous act will be deployed. In this decision, actors identify clear goals and design a strategic plan to carry out the courageous act. However, at this point, it is also possible that they decide to exit the situation. They might understand the urgency, see the value, have efficacy, and feel motivated, but ultimately fail to identify clear goals or execute an adequate plan to attain a successful outcome. They therefore exit the situation. This exit is indicated with a stop symbol in Figure 1.

The decision to engage also requires a strategy as to when to act. Imagine a risky situation in which acting with patience may be more beneficial for the sought-after noble cause than immediately acting with courage. For example, in situations that make one wonder “if that is the hill they want to die on,” in the context of a more important goal, the actor may choose not to engage immediately, despite the urge to act courageously. This choice can be exemplified by the idea of “losing a battle to win a war.” In situations like these, although the actor is not avoiding the situation, they are not immediately approaching it either. Instead, they are looking for a more appropriate time to act, despite having the necessary courage and motivation to act. Consequently, the actor may decide to wait until a later time, despite checking all the prerequisites of the process of acting courageously listed in the previous steps.

### 4.7 Action

Once the actor has decided to engage, they commit to the situation and perform the courageous act. Their act may include multiple sub-actions. For example, if, after witnessing bullying in class, a student decides to respond to the situation, they may speak up immediately, intervene in the situation, gather help, record the incident, reach out to the authorities, and/or inform parents. After the incident, they may follow up on the case, write about it in the school’s magazine, become an explicit ally of the victim, or even start a school-wide anti-bullying campaign. All of these actions are courageous ones that were triggered by witnessing the bullying behavior.

Similarly, in the case of the protests in Iran, the protestors have chosen a variety of relevant actions. These actions include arranging flash mobs, chanting slogans demanding basic human rights, blocking streets to slow down security forces, organizing sit-down strikes, symbolically burning and tearing off hijabs (women’s headscarves), dismantling public “security” cameras, chanting from rooftops and windows, dyeing fountains in blood-red colors, women symbolically cutting their hair, students boycotting classes, and professors resigning from their government jobs (Alkhaldi and Ebrahim, 2022; France 24, 2022; Luo and Umar, 2022; Reuters, 2022a,b).

Courageous actions can take a wide range of forms. They can be episodic (one-time) or frequent; simple or elaborate; mild or intense; overt or covert; subtle or explicit; and commonplace or heroic. The courageous actions can manifest through thoughts and emotions; expressed as words and visuals; and as physical acts involving gestures and steps. There can be multiple courageous actions for a particular situation, but the effectiveness of an action cannot be fully predicted in advance. Navigating such uncertainty is inherent to acting courageously (Shelp, 1984). Acting courageously often does not yield the hoped-for result. In fact, it may even yield the opposite, undesirable result, which invites a discussion on the outcomes of courage.

### 4.8 Outcomes

Courageous actions have consequences, and each action may result in a different outcome. These outcomes can be good or bad, or a little of each; positive and negative, or a little of each; desirable and undesirable, or a little of each. For example, in our previous example of Iranian protests, each individual action can result in different outcomes. Protestors’ immediate decisions have invited brutal retaliation from the government (France 24, 2022; Rajvanshi, 2022). Participating in sit-down strikes or public marches has resulted in mass arrests and detentions. Even worse, at least seven protestors have been judicially executed for disturbing public order (Fassihi and Engelbrecht, 2023). Moreover, hundreds of protestors, including children, have died while resisting the security forces (Reuters, 2022b). Regardless, the final and collective outcome of these protests might be more positive and desirable than the abovementioned deaths and arrests, where Iranian women may see a systemic change in their life circumstances through a dramatic change in Iran’s regime and legislation. However, the final outcome cannot be predicted at the moment. Analysts are seeing the unrest as an intermediate possible step toward long-term political change (Reuters, 2022b). In that sense, the current protests may serve only as an intermediate outcome and may not achieve the ultimate goals. This, in no way, undermines the courage currently demonstrated by Iranian protestors. Today’s courageous actions can serve as a stepping stone for the next actions.

### 4.9 Experience

The outcomes of one’s courageous action can shape the actors’ experience, and that experience in turn can shape their knowledge about the situation, the actions they undertook, and the results to which those actions led. This knowledge can then feed into the actors’ memory, shape their worldview, and serve as a trigger in a similar situation in the future. Additionally, as actors take opportunities to express courage, they can gain wisdom about the situation through the process, which in turn can influence further experiences (Glück and Bluck, 2013). The experience component and its allied processes thus close the loop of the processes of courage that started with a trigger. For example, as predicted by some analysts (Reuters, 2022b), the current protests in Iran may not result in an immediate dramatic change in the country’s political leadership. However, the experience of this ongoing resistance may set the Iranian people up for future fights for their rights. Courage, especially in the context of a major systemic change, can be seen as an iterative process of trigger-deliberation-action-outcome-experience. In this process, the experience gained from the previous iteration may feed into the next cycle, and the loop may continue. Eventually, it may even end in success.

## 5 Potential moderators of the process of courage

Several factors, such as personality traits, wisdom, prior experience with sociocultural norms, and demographics can influence the processes of courage.

### 5.1 Personality traits

An individual’s dominant personality traits can be predictive of how they will respond to a situation that invites courageous action. Howard and Cogswell (2019) provided evidence of the relationship between behavioral social courage and personality traits. In their study, among personal and contextual variables, grit and proactive personality were significant predictors of courageous behavior in the workplace. On the one hand, if an actor is accustomed to defying the Zeitgeist or, perhaps, is open to new experiences, they may be more inclined to take the courageous route (Sternberg, 2018). On the other hand, someone who is timid and less tolerant of adverse experiences, which can be assumed to follow courageous acts, would be less likely to act courageously. Furthermore, perceptions of risk and outcomes of risk-taking are important facets of courage. Several personality traits can influence these facets. To that end, narcissistic personality has been linked with elevated risk-taking, which has further been found to be fueled by heightened perceptions of benefits stemming from risky behaviors (Foster et al., 2009). Such risk-taking, however, is not necessarily courageous but rather may be directed solely toward self-interest.

The enlisted personality traits are only representative and not exhaustive. A detailed review of courage-relevant personality traits is out of the scope of this article. However, it would be prudent to examine different personality characteristics--dispositional, situational, and interactive--in relation to their influence on the courage process.

### 5.2 Wisdom

Wisdom is relevant to courage, as actors engaging in courage-demanding situations employ tacit knowledge in balancing their own and others’ interests, all in favor of pursuing a noble goal (Sternberg, 2024). Baltes and Staudinger (2000) defined wisdom as an expert knowledge system concerning the fundamental pragmatics of life, which include knowledge and judgment about the meaning of life and humankind’s progression toward excellence while attending to personal and collective well-being. This definition has been expanded upon by other scholars to include more specific characteristics, including intellectual humility, recognition of uncertainty and change, consideration and integration of different perspectives, self-regulation, altruism and moral maturity, openness and tolerance, concern for others, reflective attitude, cognitive ability, insight, and real-world skills (Sternberg, 1998, 2019, 2024; Bluck and Glück, 2005; Grossman, 2017; Karami et al., 2020). A comprehensive review of wisdom research is beyond the scope of this article (see Sternberg and Glück, 2021, for a review). However, it is crucial to acknowledge the aspect of integration and dynamic balance cuts across the various wisdom models mentioned above.

An act would not be deemed courageous if it did not achieve the integration of the various aspects of wisdom. For example, someone could use their creativity and knowledge, but not their common sense and lack of concern for others to produce an action that might be more reckless and irresponsible than courageous. Imagine if the Civil Rights Movement in the USA of the 1960s resorted to violence and burning down public property. Such a rash and heedless act would have enabled the government and opposing civilians to crush the resistance, using brute force. Violence would jeopardize the safety of the protestors and undermine the entire movement as well. Using wisdom, therefore, becomes important as actors plan their courageous actions.

### 5.3 Experience with sociocultural norms

An actor’s personal experience with social and cultural norms can also have an impact on their willingness to act courageously in the face of such norms. Protests in Iran are a prime example of how people act courageously when socio-cultural norms act against a subgroup of people. Women in Iran are protesting against the norm that women should be disciplined for not wearing the Hijab (headscarf) that is mandated by the state. The protesting women are opposing this patriarchal and fundamentalist norm, which deprives them of freedom of choice. Mahsa Amini’s death, therefore, has triggered the women in Iran to embrace their womanhood and fight against the regime. Women are burning head scarves and cutting their hair in public as a symbol of an attack on their identity and resistance to unreasonable social norms (Luo and Umar, 2022).

Courage may be related to other cultural dimensions (Howard and Cogswell, 2019), such as (a) power distance, or the extent to which less powerful members of groups accept and expect unequal power distributions; (b) uncertainty avoidance, or people’s level of comfort with unstructured situations; (c) collectivism–individualism, or the extent to which people in a society are integrated into groups for which they bear a sense of responsibility; (d) humane orientation, or the extent to which people in society value the prosocial treatment of others; and (e) assertiveness, or the extent to which people in a society are assertive (Hofstede, 2011).

### 5.4 Demographics

Demographic factors such as gender and age also can influence the processes of courage (Howard and Cogswell, 2019). In particular, courage has been traditionally treated, to some extent, as a gendered concept--men are expected to exhibit courageous actions more than women (Pury and Lopez, 2010). Although there may be no real gender differences in courage, gender may still influence the decision-making involved in courageous action through social conditioning. Similarly, age can significantly influence courage. Specifically, in workplace settings, employees may become more courageous as they spend more time in an organization (Howard and Cogswell, 2019).

## 6 Discussion

In this article, we have argued that courage is often a deliberate process. We have proposed a conceptual model that describes what steps may be involved in courage, what kinds of factors might lead people to be more or less likely to display courage, and what assessments people are likely to make when they choose to act courageously or not. In this section, we discuss the potential empirical tests of our model and describe the future directions that can emerge from our proposed process model of courage.

### 6.1 Potential tests of the model and future directions

Our article introduces a dual-process model of courage, aimed at understanding courage as a process. Empirical research is necessary to validate and refine this conceptual model. This research would involve validation, exploration and measurement, and application of the proposed process and its components. Below, we present only preliminary directions, and actual research could take numerous other forms. The objective of presenting these directions is to initiate a discussion on future research on this model.

#### 6.1.1 Direction 1: validation

Validation is an essential step in establishing the credibility and utility of our model. A first assessment to yield evidence of the validity of our model might consist of testing the alignment of our model with real-world behaviors and outcomes related to courage. To assess the content validity of the proposed model, for example, researchers could create checklists and scales with items outlining behaviors associated with each step of our model. A panel of experts in the study of courage, decision-making, and related areas could be asked to judge the relevance and representativeness of each of the items and the components. Then researchers can examine the factor structure of the instrument.

To gather validity evidence about the utility of the model, researchers could conduct interviews regarding courageous actions that key participants have witnessed or undertaken. Researchers could use the think-aloud strategy to ask participants to verbally recreate the perceived sequence of events. Similar to a study by Schilpzand et al. (2015), the collected data could be analyzed following the proposed model. The researchers asked participants to recount an event in which courage was displayed. Then, participant stories were used to assess components of courage in the researchers’ proposed model (Schilpzand et al., 2015). Results from the think-aloud could help researchers evaluate the salience or non-salience of the assessment points in our model. By comparing participants’ responses to the model’s expected outcomes; a closer match would indicate higher model accuracy.

#### 6.1.2 Direction 2: exploration and measurement

Exploration involves further investigating and examining the relationships among factors that influence the processes of courage. Our model proposes that personality traits, wisdom, social norms, and demographics moderate the courage process. In essence, courage is a personality x task x situation interaction, as are many other psychological phenomena (Sternberg et al., 2023). Whether a given person will be courageous will depend on the task they confront (e.g., saving a cat in a burning building vs. protesting for civil rights) and the situation in which they confront that task (e.g., the result might be disapproval of friends vs. life imprisonment). Courage always depends on intrapersonal variables, but also on the tasks at hand and the context in which they are presented.

Studies such as Howard and Cogswell’s (2019) have explored some of these factors as predictors of courage in the context of social courage. Researchers could measure the extent to which motivation, wisdom, personality traits, and demographic variables influence courageous behaviors. Specifically, exploration efforts could lead to identifying the characteristics and contexts in which people are more likely to engage in courageous action.

Another future direction of this research would include developing a scale to better understand situated courageous actions and how people approach challenging situations. Participants can be asked to select the extent to which they engaged in behaviors and thought processes related to our model. For example, using a Likert-type scale, to what extent they acted instinctively and instantaneously; assessed the situation as to whether it required an immediate response; weighed to what extent the situation was personally meaningful and how high the stakes were for the individual; engaged in moral considerations and risk assessment involved in the approach-avoidance conflict; analyzed potential outcomes and perceived likelihood to succeed; balanced interests of self and others; and reflected on the action and the whole situation. Our process model may be applied as a theoretical framework for this scale-development study.

#### 6.1.3 Direction 3: application

The test of application consists of identifying the extent of the model’s usefulness. Our model aims to be a model of general courage beyond existing models, which focus primarily on the workplace (e.g., Schilpzand et al., 2015). Researchers could apply the process model of courage to different contexts and domains to elicit behaviors related to courage. According to our proposed model, a courage process involves four assessments, namely, (a) immediacy of the situation; (b) meaningfulness, relevance, and value to the actor; (c) adequacy of efficacy to act; and (d) decision to act with courage. Participants would be given a set of open-ended problems that require courageous action. Some examples might include (a) saving someone in a building on fire, (b) saving someone on a battlefield, (c) speaking the truth when one’s job is at risk, (d) speaking out publicly against a demagogue, and so on. Participants would be asked to elaborate on their proposed solution. Researchers can examine the extent to which the participant solution (a) identifies the morally right thing to do in the situation, (b) realizes that one is taking an enormous personal risk, (c) realizes that one may not succeed, and so on. The prediction is that plausible solutions will match the assessment points and thought processes outlined in our model.

Finally, the model could be assessed against existing models of courage. For example, researchers could evaluate the conceptual clarity, empirical evidence, and predictive power, as well as the scope and applicability of our model compared to existing models. Researchers could assess courageous acts (actual or vignette examples) using multiple models, contrasting the strengths and weaknesses of each model in explaining the courageous acts. The goal of this assessment would be to further strengthen our model by pointing out opportunities to reduce conceptual overlaps, refining strategies to gather new data, or suggesting applications to enhance the courage process.

Overall, future research on this model should aim to extend the current understanding of the multidimensional construct of courage and refine our search for the processes involved in courageous behavior. This model can be seen as a framework for understanding the nature and underlying decision-making processes of courageous behavior. Using this process model, people could be asked to respond and reflect on each step of the flowchart. Perhaps this model could be used to understand a variety of outcomes, such as people’s perceptions of courage, to identify triggers or situations that require courage, to illustrate processes and strategies by which courage can be elicited, and to ascertain what it takes to act courageously.

## 7 Conclusion

Courage is often part of the way we make choices about what is important, what to do, and how to go on in life in ways we view as worthwhile. Maintaining a view that courage is often a deliberate process, we have proposed a model that describes what steps may be involved in courage, what kinds of factors might lead people to be more or less likely to display courage, and what kinds of assessments people are likely to make when they choose to act courageously or not. We have shown how courage depends not only on the person but also on the particular tasks the person confronts and the situational context in which they are presented. The proposed dual-process model of courage can, thus, serve as a stepping-stone to understanding how to navigate decision-making in the face of difficult situations demanding courageous responses. Ultimately, navigating this process of courage is key to meeting many of the demands and challenges of our troubled world.

## Data availability statement

The original contributions presented in the study are included in the article/supplementary material, further inquiries can be directed to the corresponding author.

## Author contributions

AC: Conceptualization, Investigation, Methodology, Project administration, Supervision, Writing – original draft, Writing – review & editing, Visualization. FP-M: Conceptualization, Writing – original draft, Writing – review & editing. MG: Conceptualization, Writing – original draft, Writing – review & editing. ZB: Writing – original draft, Writing – review & editing. GF: Writing – original draft, Writing – review & editing. RS: Conceptualization, Supervision, Writing – original draft, Writing – review & editing.
